# Bi-directional effects of vitamin B_12_ and methotrexate on *Daphnia magna* fitness and genomic methylation

**DOI:** 10.1038/s41598-017-12148-2

**Published:** 2017-09-19

**Authors:** Fitore Kusari, Alan M. O’Doherty, Nikolas J. Hodges, Marcin W. Wojewodzic

**Affiliations:** 1 University of Birmingham, School of Biosciences, Edgbaston, Birmingham B15 2TT, UK; 2 School of Agriculture & Food Science, University College Dublin, Belfield, Dublin 4 Ireland

## Abstract

Here we interrogated, using three separate but complementary experimental approaches, the impact of vitamin B_12_ availability and methotrexate exposure on *Daphnia magna*, which we hypothesised should have an opposite effect on One carbon metabolism (OCM). OCM is a vital biological process supporting a variety of physiological processes, including DNA methylation. Contrary to mammalian models, this process remains largely unexplored in invertebrates. The purpose of this study was to elucidate the impact of OCM short-term alteration on the fitness and epigenome of the keystone species, *Daphnia*. We used maternal age at reproduction, brood size and survival rates in combination with DNA methylation sensitive comet assay to determine the effects of vitamin B_12_ or MTX on fitness and the epigenome. Vitamin B_12_ had a positive influence on *Daphnia* fitness and we provide evidence demonstrating that this may be associated with an increased level of genome-wide DNA methylation. Conversely, exposing *D. magna* to MTX negatively influenced the fitness of the animals and was associated with loss of global DNA methylation, translating in decreased fitness. These results highlight the potential importance of OCM in invertebrates, providing novel evidence supporting a potential role for epigenetic modifications to the genome in *D. magna* environmental adaptability.

## Introduction

In recent years the importance of the water flea, *Daphnia*, as a model organism for studying toxicological genomics, ecology and evolution has been highlighted by a number of groups^1–4^. This freshwater planktonic microcrustacean has also been proposed as a model organism for studying developmental programming and epigenetics, as clonal lines contain genetically identical but phenotypically divergent individuals^5^. Although *Daphnia* has been identified as an important species for epigenetics research and DNA methylation exists in its genome^6^, there are still very few studies in the literature^7,8^ connecting directly the DNA methylation status with its ecological function.

*Daphnia* species are cyclic parthenogens that are capable of reproducing sexually or asexually, depending on their environment, under favourable environmental conditions they reproduce asexually, developing apomictically from diploid oocytes^9^. Sexual reproduction is favoured under more challenging or restrictive environments experiencing variations in photoperiod, decrease in temperature, increased population density and/or food shortages^9,10^. *Daphnia* spp. have also been shown to exhibit phenotypic plasticity in response to other environmental cues, such as the formation of helmets and neckteeth when exposed to predators and tolerance adaptation to increased levels of salinity^5,11,12^. Recently, *Daphnia* has been proposed as a model organism to study Environmental Epigenetics^13^.

DNA methylation, a vital epigenetic modification involved with gene regulation, has been extensively studied in mammals for nearly two decades^14^. It is widely regarded that DNA methylation in mammals is susceptible to external environmental cues and that these changes may result in phenotypic alterations^15–17^. Conversely, the genomic levels and role of DNA methylation has only recently begun to be unraveled in invertebrate species^18^. Most recently, analysis of gene body methylation in two species of *Daphnia* revealed both highly methylated and unmethylated gene bodies; with the level of methylation being associated to gene family size^7^. Furthermore, differential gene body methylation (specifically exonic methylation) has been shown to occur in *Daphnia* exposed to the toxic cyanobacterium *Microcystis aeruginosa* ^8^.

DNA methylation reactions are catalyzed by DNA methyltransferases (DNMTs), which utilize methyl groups supplied by OCM^19^. Folate and methionine metabolism constitute the OCM pathway^20^. S-adenosylmethionine (SAM), the universal methyl donor for DNA, RNA and histone methylation reactions, is generated through OCM^21^. Intracellular availability of SAM is dependent upon the availability of several key nutrients and vitamins such as methionine, vitamin B_6_, vitamin B_12_ and folate^20^. Diets with suboptimal and/or supraphysiological levels of these key players can impair methyl group availability, resulting in aberrant DNA methylation patterns^22^. While evidence linking nutritional deficit and epigenome alterations (aberrant DNA methylation) is abundant in mammals^22–27^ these studies are currently lagging behind in aquatic invertebrate species. Although there is evidence in *Daphnia* spp. demonstrating that reproductive performance is linked to dietary vitamin B_12_^28^. In addition, links between nutrition, DNA methylation and caste determination have been identified in social insects such as honeybees and ants^29,30^. In the invertebrate, *Caenorhabditis elegans*, B_12_ is essentially required for growth and its genome contains a full complement of homologs of mammalian B_12_-related metabolic pathways^31^; suggesting a possible role for B_12_ in OCM in other invertebrate species. To increase our understanding of invertebrates, with respect to vitamin B_12_ regulation and OCM, using *D. magna* as a model organism, we raised three main questions to test our hypothesis that environmentally induced phenotypic changes, observed in *Daphnia* spp., are associated with underlying epigenetic alterations to the genome: (i) Do *Daphnia* respond to quick dietary shifts (acute exposure) in vitamin B_12_ concentration, being a key component of one-carbon metabolism and DNA methylation? (ii) How do animals respond when food conditions are not changed, but OCM is perturbed using methotrexate (MTX)?, a known antagonist of one-carbon metabolism in mammals and invertebrates^32–34^ (iii) Do vitamin B_12_ supplementation or MTX exposure differentially impact DNA methylation of daphnids?

To address these questions, we first subjected *D. magna* to culture under concentration gradients of vitamin B_12_ or methotrexate (MTX) and recorded their fitness. We then assessed global DNA methylation levels using an easily accessible, medium-resolution DNA methylation comet assay developed for this purpose^35^.

## Materials and Methods

### Sample preparation

**Figure 1:**
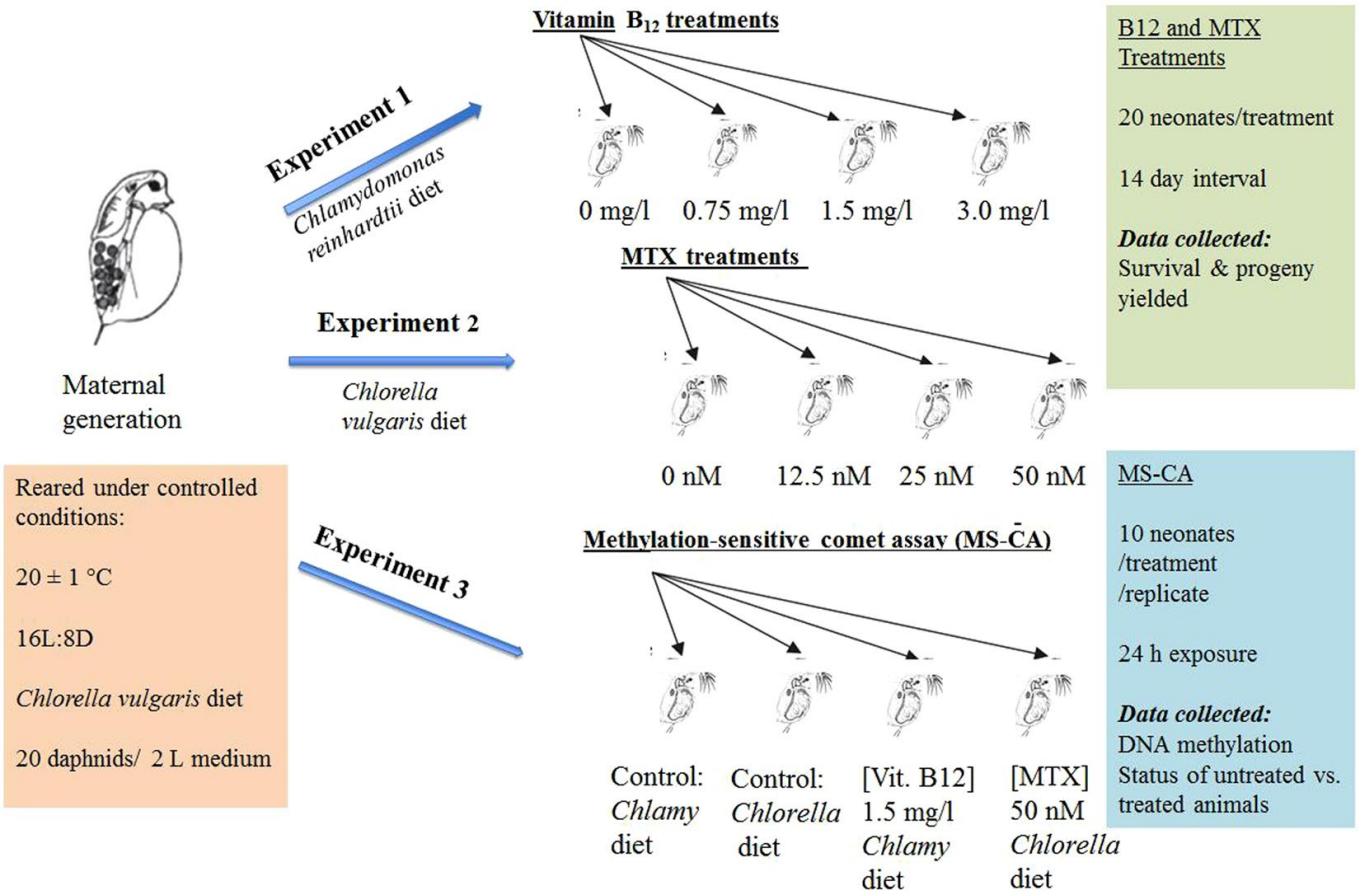
Experimental Design. A scheme showing overview of three experiments including chronic exposure— vitamin B_12_ and methotrexate (MTX) treatments and methylation sensitive comet assay (MS-CA) in *Daphnia*.

*Daphnia magna* clones (Straus, Bham2 clone housed in Birmingham, UK) were cultured for several generations prior to the experiment under standardized conditions according to the US Environmental Protection Agency guidelines (EPA-821-R-02–012) to minimize maternal effects. Unless stated, all reagents were supplied by Sigma–Aldrich, Dorset, UK. During initial culture and acclimation 20 daphnid neonates (≤24 h) were maintained in 20 °C ± 1 °C charcoal and UV filtered and aerated borehole water and fed daily, *ad libitum*, with 2.7 × 10^4^ *Chlorella vulgaris* cells per daphnid for 7 days and 5 × 10^5^ cells/daphnid, thereafter. Culture media was replaced weekly and culture vessels were kept under a summer photoperiod (16:8 h – light:dark). In the first experiment, investigating the acute effects of vitamin B_12_ on *D. magna*, *Chlamydomonas reinhardtii* algae (vitamin B_12_ deficient strain) were used as a food source as they are able to grow in vitamin B_12_ deficient media^36,37^. *Chlamydomonas* (+) 15–2040 (Carolina Biological Supply Company, USA) was prepared by inoculation in modified Bolds Basic Medium (Bold 1949, Bischoff and Bold 1963) for 5 days at 25 °C with permanent light. The *Chlamydomonas* was harvested by centrifugation at 3000 × g for 15 min and resuspended in borehole water. In the second experiment investigating the impact of MTX on *D. magna*, Daphnids were fed another chlorophyte, *Chlorella vulgaris* (grown in the same medium, light and temperature conditions as *Chlamydomonas*). The overall experimental design is outlined in Fig. 1.

### Experimental design

#### Experiment 1 – evaluating the effect of vitamin B_12_ availability on overall *D. magna* fitness

Neonates (≤24 h) from stock cultures were switched to *Chlamydomonas*, a B_12_ deficient diet and exposed to four vitamin B_12_ culture conditions over 14 days; high (3.0 mg L^−1^), medium (1.5 mg L^−1^), low (0.75 mg L^−1^) and control-deficient (0 mg L^−1^ vitamin B_12_ deficient). By increasing media concentrations of B_12_, on a background diet deficient in B_12_, we hypothesised that any improvement of fitness observed, would be due to increasing availability of dietary B_12_ – required for OCM. The selection of vitamin B_12_ concentrations was based on a preliminary acute experiment (48 h) indicating that animals survived and displayed no immobilization at concentrations of B_12_ ranging from 0 to 6.0 mg L^−1^ (data not shown). For chronic exposure treatments, twenty *D. magna* neonates (from separate mothers) (≤24 h), from the second brood, were cultured individually in 80 ml of test solution per treatment. During the first 6 days of culture Daphnids were fed 3 × 10^4^ *Chlamydomonas* cells per day and on days 7–14 the concentration was increased to 6 × 10^6^, to fulfil their increased metabolic requirements. The culture media was renewed every second day, during which time organismal survival and reproductive capacity (number of progeny produced) was recorded.

#### Experiment 2 – evaluating the effect of methotrexate on *D. magna* fitness

An initial test of different methotrexate (MTX) concentrations (50 nM, 25 nM, 12.5 nM, 6.25 nM, and control (lacking MTX)) on *D. magna* was performed for 48 h. All of the Daphnids survived for 48 h and no immobility was determined by stereomicroscopic examination. Based on the observation that low-dose MTX had no impact on survival or mobility, treatment groups containing 50 nM, 25 nM and 12.5 nM and a control group lacking MTX were investigated. These low-dose concentrations were selected based on previous findings by Wang *et al*.^33^. For each group, 20 Daphnids were cultured individually in 80 ml media over a 14 day exposure period. *Daphnia* were each fed 2.7 × 10^4^ *Chlorella vulgaris* cells for 6 days, and 5.5 × 10^4^ cells of algae per *Daphnia* for days 7–14. Every second day the media was renewed, survival, the number of live progeny and the time from the release of the first brood and second brood by each daphnid was recorded. Neonates were removed during this time to prevent their exposure to MTX and consumption of algae intended for the adult *Daphnia*.

#### Experiment 3 - Methylation sensitive comet assay (MS-CA)

Sensitivity of *Daphnia* spp. to environmental insults has been reported previously^38^ and, typically, phenotypic changes are observed rapidly^39–41^. We hypothesised that rapid eco-response of *Daphnia* may be mediated through alterations to their epigenome. To address this, DNA methylation, in the context of CCGG sequences throughout the *Daphnia* genome, was assessed over a short period of time (24 h) following dietary changes (i.e. vitamin B_12_ supplementation) or in the presence of xenobiotics (MTX). Global DNA methylation patterns were determined using comet assay combined with methylation sensitive DNA digestion^35^, adapted by our group for use in *Daphnia* (MS-CA). This method is cost effective, allows for processing a large number of samples simultaneously and does not require advanced bioinformatics; thus offering a rapid method for screening DNA methylation alterations. However, the method has a disadvantage in terms of resolution – identification of loci specific alterations to DNA methylation is not possible. For the MS-CA experiment, 10 second brood *D. magna* juveniles (≤24 h) were exposed to (1) 1.5 mg L^−1^ vitamin B_12_ or (2) 50 nM MTX for 24 h and respective controls. Animals in the vitamin B_12_ group were fed 3 × 10^4^ *Chlamydomonas* cells per daphnid and animals in the MTX group fed 2.7 × 10^4^ *Chlorella vulgaris* cells per daphnid. Experiments were performed in duplicate alongside control conditions lacking either vitamin B_12_ or MTX. After 24 h, 10 whole juvenile daphnids from each treatment and control group were sampled to eliminate tissue selection bias of the *Daphnia* and/or the presence of eggs in the brood pouch that could lead to potential variations in DNA methylation. Animals were placed in 1 ml phosphate buffered saline (PBS) containing 20 mM EDTA and 10% DMSO, as described previously^42^. Samples were homogenised by continuous pipetting for 2 min at 4 °C then centrifuged at 5000 rpm for 5 min and the supernatant removed. Homogenised pellets were resuspended in a 100 µl solution of 0.7% low melting point agarose (Promega, Southampton, UK) and spread onto microscope slides pre-coated with 1% standard agarose and the agarose allowed to set by cooling slides on a cold metal block at 4 °C. Next, slides were submerged in ice cold lysis buffer containing 10 mM Tris, 100 mM EDTA, 2.5 M NaCl, 10% DMSO and 1% Triton X-100 for 1 h to prepare nucleoids. Exposed nuclei were digested for 30 min at 37 °C in 50 µl reactions containing 1 x CutSmart buffer and 10 U HpaII, MspI (New England Biolabs, Hertfordshire, UK) or no enzyme, respectively. Prior to enzymatic digestion, slides were saturated in endonuclease buffer (10 mM Tris–HCl, 10 mM NaCl, 1 mM β-mercaptoethanol and 2 mM EDTA) for 10 min to create optimal conditions for enzyme catalytic activity. Digested nuclei were exposed to alkali conditions (1 mM Na_2_EDTA and 300 mM NaOH) for 20 min at 4 °C, followed by electrophoresis at 300 mA, 0.8 V/cm at 4 °C in the dark for 20 min. After electrophoresis, the slides were neutralised with 0.4 M Tris-HCl buffer, pH 7.5 for 15 min. DNA was fluorescently labelled with a 1:10,000 dilution of SYBR gold for 1 hour (Life Technologies ltd, Paisley, UK). Comet slides were analysed using a fluorescence microscope (Zeiss, Axiovert) equipped with a 515 to 560 nm excitation filter and a barrier filter of 590 nm. at 400 X magnification using a 40 X oil immersion lens and Komet IV image analysis software (Perceptive Instruments, Bury St Edmunds, UK). Measurements of percent (%) tail DNA of 50 random comets per slide were taken and the median value used as the unit for statistical analysis as recommended by Duez *et al*.^43^. DNA digestion was quantified by measuring the percentage of DNA migrating from the comet head (% tail intensity). For all samples, genome methylation was calculated using the formula [(100–HpaII\MspI × 100) – control], where HpaII\MspI represent are the average percentage tail DNA resulting from HpaII- and MspI-digested nucleoids and^35^. Control (slide no enzyme) was subtracted to account for the basal DNA damage prior to enzymatic digestion. As an internal control, the skin cancer cell line (HaCaT) with known methylation level, was also included.

### Statistical Analysis

Malthusian parameter (r) was calculated as a metric of performance following supplementation with vitamin B_12_ or exposure to MTX according to the formula 1 = (e^rt1^l_t1_m_t1_) + (e^rt2^ l_t2_m_t2_), where t_1_ = time to first brood, t_2_ = time to second brood, m = brood size and l = survivorship that was set to 1 as mortality was not observed during exposures. Small r was iterated with left part of the equation set to 1 for comparability. A higher value of this parameter suggests a fitter organism. The environment for statistical computing “R” version 3.1 (R development Core Team, 2008) was used for statistical analysis. The effect of vitamin B_12_ and MTX on fitness was analysed separately by generalized linear model (glm) with the treatment concentration as the explanatory variable. The models did not show any significant sign of violation of the assumption for the test (data not shown). The effects of different treatments (food regimes) were compared using generalized linear model. MS-CA was also compared by glm, with treatment as the explanatory variable. Two independent experiments were performed so that we were able to test if the experiment had an effect.

## Results

### Vitamin B_12_ supplementation is associated with improved *D. magna* fitness

**Figure 2:**
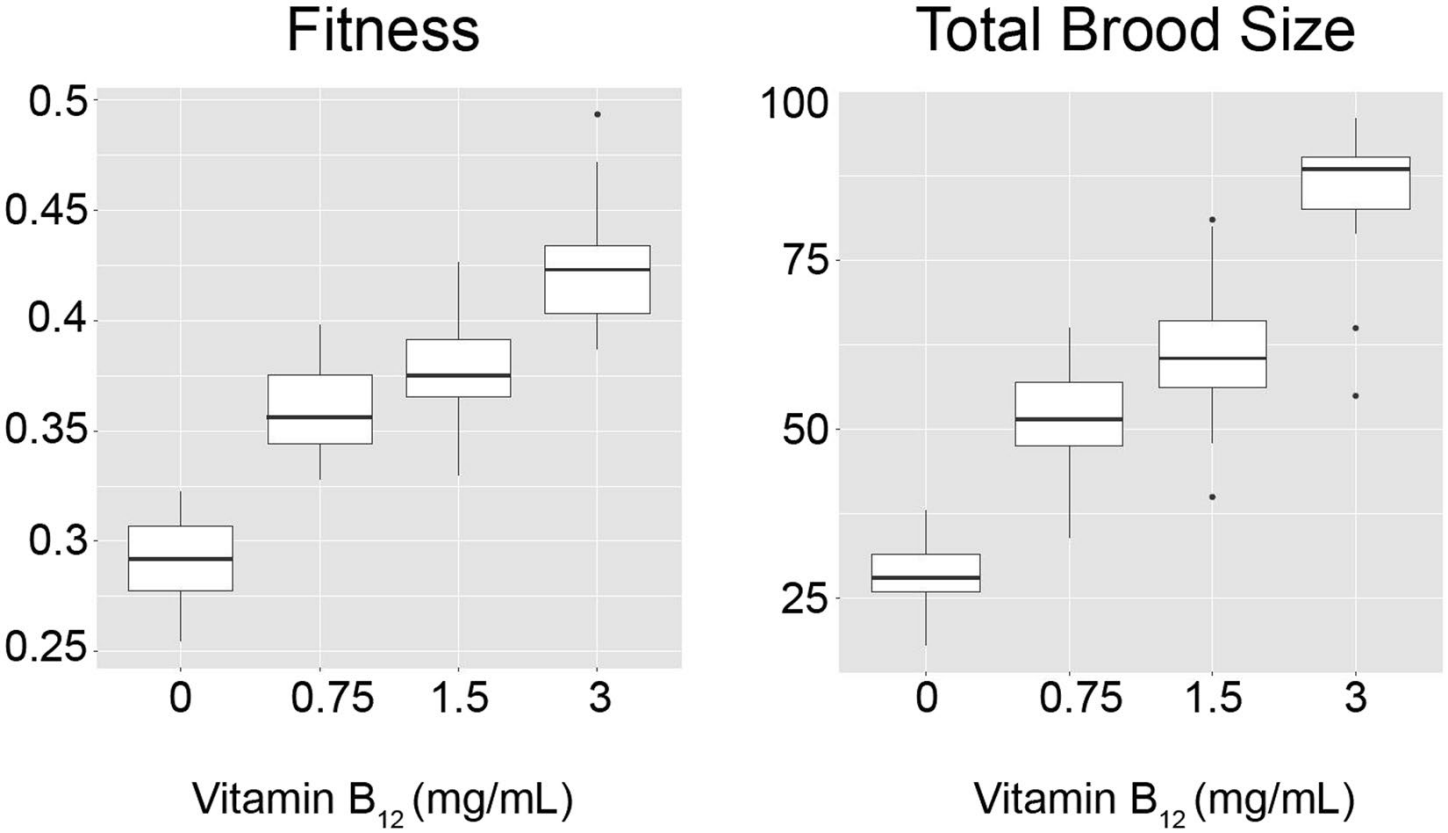
Impact of vitamin B_12_ availability on *Daphnia magna* fitness and reproductive performance. Left panel – Box-plot of fitness versus vitamin B_12_ concentration. Right panel – total brood size relative to vitamin B_12_ concentration. First and third quartiles presented with a vertical line inside to indicate the mean value respectevely for all figures.

Life history traits for 80 *D. magna* exposed to different concentrations of vitamin B_12_ (control – no supplemental vitamin B_12,_ low – 0.75 mg/L, medium– 1.5 mg/L and high – 3.0 mg/L) were recorded over a 14 day period to determine the relationship between vitamin B_12_ availability and *D. magna* fitness. These traits enable population growth rate calculations, which translate into fitness. Age at first reproduction, age at second reproduction, size of first brood, size of second brood and survival rate were recorded. Generalized linear model analysis revealed that for every 1 mg L^−1^ increase of vitamin B_12_, *D. magna* fitness significantly increased by 0.039 (*P* ≤ 0.001) (Supplementary Table 1). Average fitness for each treatment group was as follows: 0.26 ± 0.09 (0 mg L^−1^), 0.36 ± 0.02 (0.75 mg L^−1^), 0.37 ± 0.03 (1.5 mg L^−1^) and 0.42 ± 0.02 (3.0 mg L^−1^) (Fig. 2). Progeny yielded by *D. magna* was also proportional to the concentration of vitamin B_12_ that they were exposed to in the media. In the 0 mg L^−1^ group there were a total of 33 juveniles, increasing by 17 for every mg of vitamin B_12_ added per litre of media (Supplementary Table 2 and Fig. 2). Data related to the impact of vitamin B_12_ concentration on the size of the first and second broods is outlined in Supplementary Figure 1.

### Reduction of Daphnia magna fitness following exposure to methotrexate

**Figure 3:**
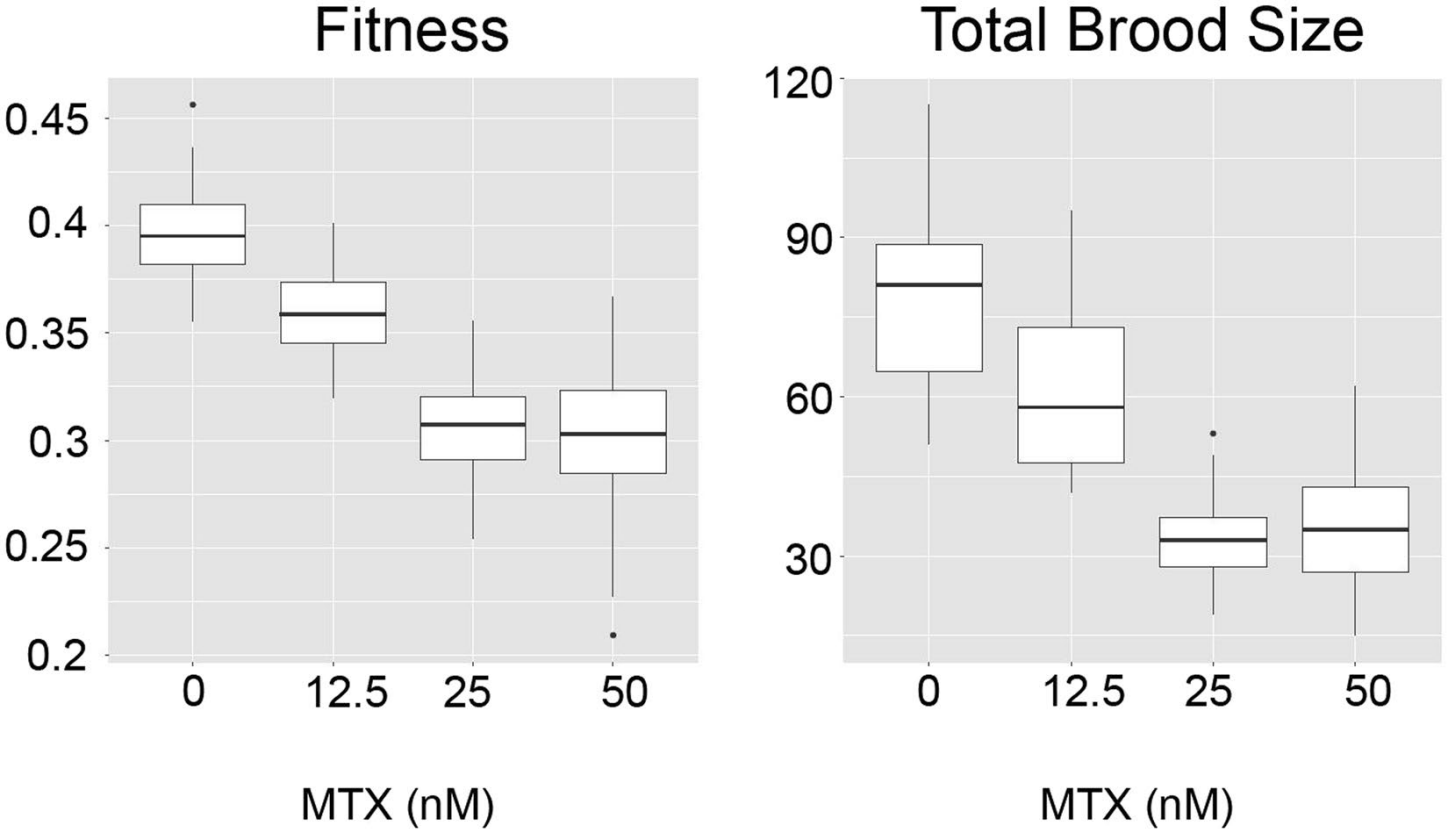
Impact of MTX exposure on *Daphnia magna* fitness and reproductive performance. Left panel – Box-plot of fitness versus MTX concentration. Right panel – total brood size relative to MTX concentration.

A similar experimental strategy as above was employed over a 14-day period to examine the effects of MTX exposure on *D. magna* fitness. Fitness was compared across four experimental groups maintained under the following media conditions; control animals (no MTX), 12.5 nM, 25 nM and 50 nM MTX using the generalized linear model. It was observed that for every 1 nM increase in MTX concentration *D. magna* fitness decreased by 0.002 and it was statistically significant compared to the animals that were not exposed to MTX (*P* ≤ 0.001) (Supplementary Table 3). *D. magna* mothers that were not exposed to MTX had the highest fitness and increasing levels of MTX was proportional to decreased fitness; average fitness for each treatment group was as follows: 0.39 ± 0.03 (0 nM), 0.36 ± 0.02 (12.5 nM), 0.30 ± 0.02 (25 nM) and 0.25 ± 0.12 (50 nM) (Fig. 3). A marked decline in reproductive performance was observed along the MTX concentration gradient – total number of offspring (first and second brood combined) in the unexposed group was equal to 72 and decreased by 8.9 for every 10 nM increase of MTX in the media (Supplementary Table 4 and Fig. 3). Data related to the impact of increasing concentrations of MTX on the size of the first and second broods are outlined in Supplementary Figure 2.

### Vitamin B_12_ availability and methotrexate (MTX) exposure impact global DNA methylation levels

**Figure 4:**
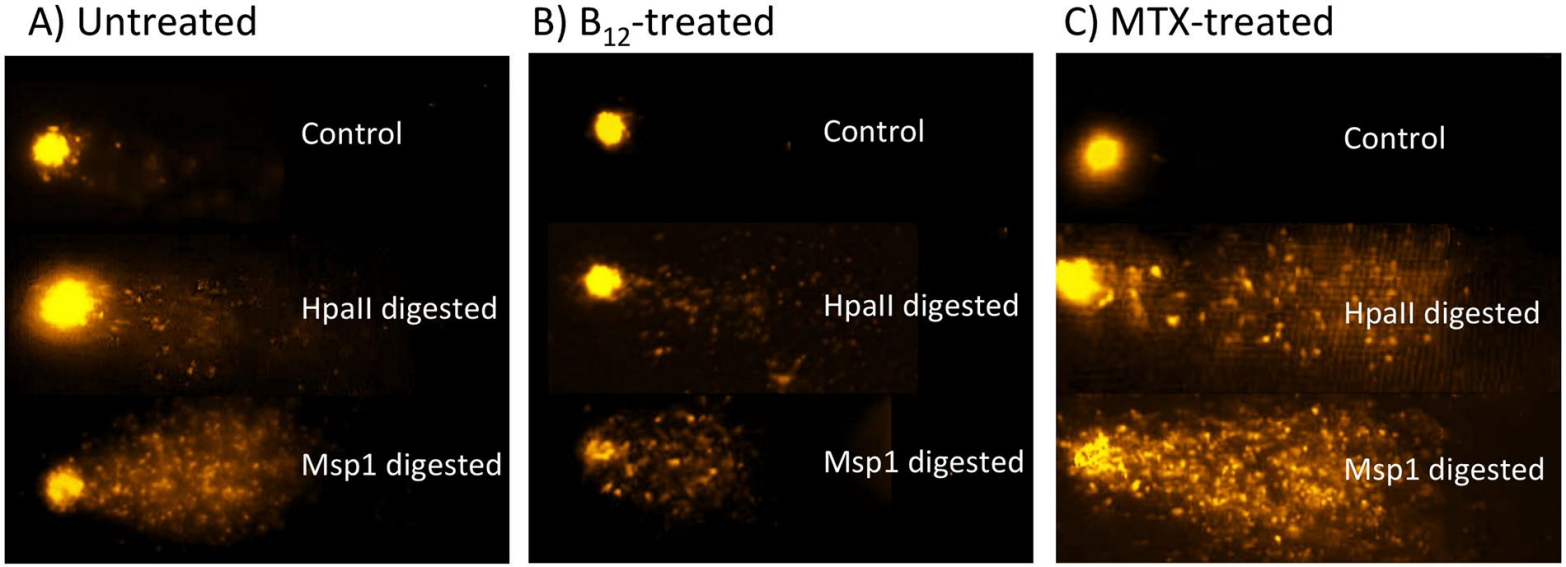
Methylation sensitive comet assay (MS-CA). The observed comet tails represent double-strand DNA breaks, introduced by the isoschizomeric enzymes MspI and HpaII; in the presence of an electric field, the HpaII- and MspI-digested DNA migrates faster, compared to the significantly slower migration of the undigested DNA. Digestion of the control (Chlamy) and treated (vitamin B_12_ and MTX) samples resulted in a tail DNA increase for MTX and decrease for vitamin B_12_, relative to the intact DNA in the comet head of the untreated controls – indicative of decreased DNA methylation levels in MTX-treated animals and increased DNA methylation in vitamin B_12_-treated animals.

**Figure 5:**
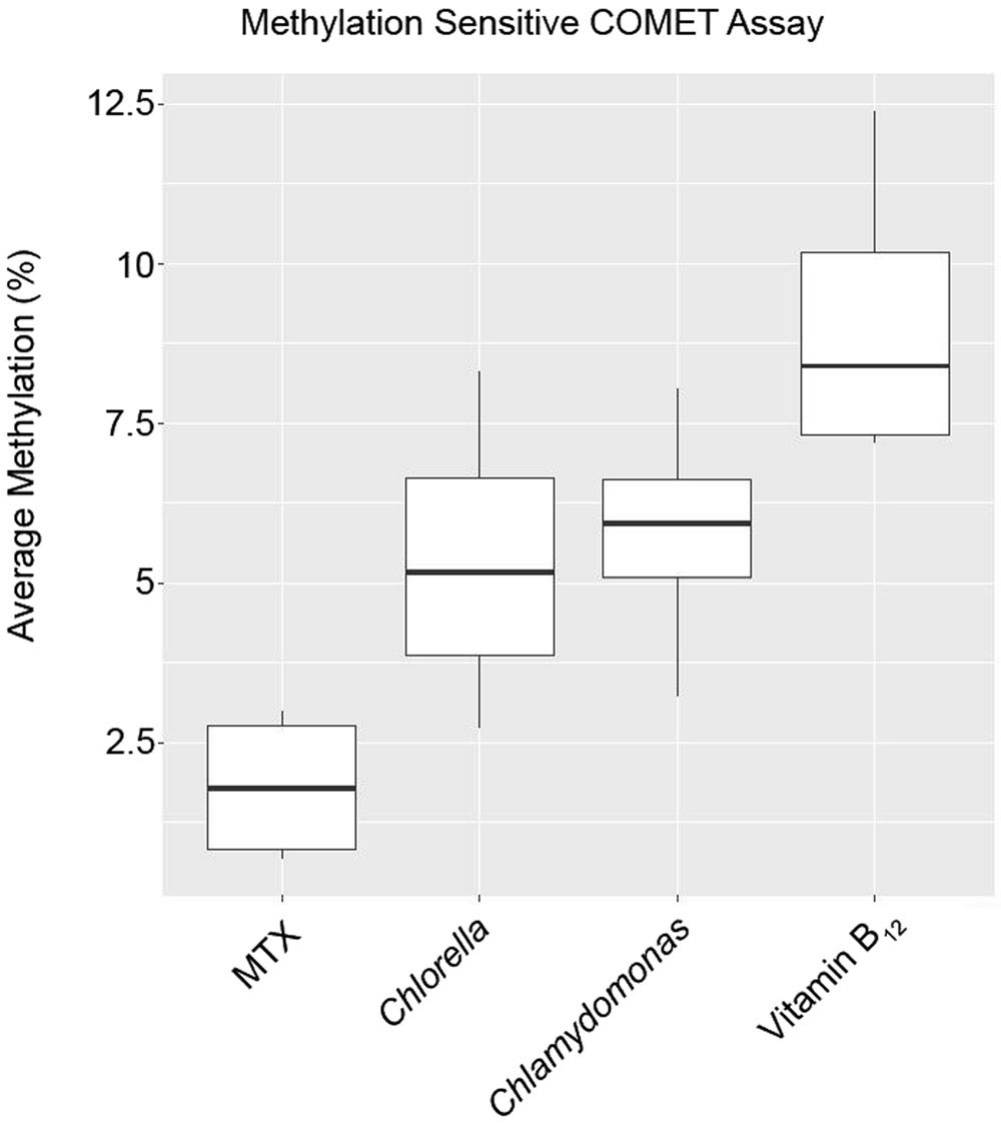
Global methylation profiles of *Daphnia magna* supplemented with vitamin B_12_ or exposed to MTX. The y-axis represents percentage DNA methylation levels following methylation-sensitive comet assay. The x-axis denotes the *Daphnia* sample groups; vitamin B_12_-treated, MTX-exposed and untreated controls (*Chlamydomonas* and *Chlorella*). A box and whiskers are present for each sample.

We next used methylation sensitive comet assays (MS-CA) to evaluate the effect of vitamin B_12_ supplementation and MTX exposure on genome wide methylation levels (Fig. 4). Animals were exposed to media supplemented with either 1.5 mg/L vitamin B_12_ or 50 nM MTX for 24 h alongside daphnids maintained in non-supplemented media. The average level of methylation for B_12_-treated animals was 9.09 ± 2.4%, which was significantly larger than that observed for the control animals; *Chlamydomonas*, 5.34 ± 2.4% and *Chlorella* 5.8 ± 1.9% (Table 1 and Fig. 5). MS-CA analysis comparing untreated control animals to MTX-exposed animals revealed that a 24 h exposure of *D. magna* to media supplemented with 50 nM MTX significantly reduced global DNA methylation, average methylation in MTX-treated animals was 1.81 ± 1.2% (Table 1 and Fig. 5). Summary statistics of vitamin B_12_ vs control and MTX are outlined in Table 2. The average level of methylation in Human keratinocytes (HaCaT), with a documented degree of DNA methylation^44^, was observed to be 48.7 ± 1.2% for two independent experiments (Supplementary file 3). This level of methylation is in agreement with data previously published using HaCaT cells^44^; thus serving as an internal control validating the MS-CA method.

**Table 1:** Global DNA methylation levels in treated and untreated *Daphnia magna*

Treatment group	Mean (%)	Standard Deviation
Vitamin B_12_	9.09	2.4
Methotrexate	1.81	1.2
Chlamy-Control	5.34	2.4
*Chlorella*-Control	5.78	1.9
Skin (HaCaT)	48.7	1.2

Data presented in this table shows the mean global methylation level (±standard deviation (SD)) of treated and untreated *D. magna* in two independent experiments, in duplicates. Mammalian skin cell line (HaCaT) with known DNA methylation levels, served as an internal control for MS-CA and DNA methylation calculations.

**Table 2:** Summary statistics of MS-CA performed by generalized linear model (Vitamin B_12_ vs. control and MTX). Null deviance was 157 on 15 degrees of freedom and AIC was 73.8.

	Value	Standard error	t-value	p-value
Intercept	10.96	1.82	6.03	<<0.001
Methotrexate	−7.28	1.42	−5.11	<<0.001
Chlamy-control	−3.75	1.42	−2.63	0.023
Chlorella-control	−3.31	1.42	−2.32	0.040
Experiment	−1.24	1.01	−1.23	0.24

glm revealed that MTX exposed animals display a 7.28% decrease in global DNA methylation compared to *Daphnia* supplemented with 1.5 mg/L vitamin B_12_. A similar trend was observed in control animals fed with *Chlamydomonas* (Chlamy) and *Chlorella* (with decreases of 3.78% and 3.31% in DNA methylation, respectively, relative to vitamin B_12_).

## Discussion

To date, the ecological stoichiometry field has largely focused on other limitations such as nitrogen, phosphorous, zinc or iron^45–47^. However, the potential role of epigenetic mechanisms in ecological stoichiometry has yet to be evaluated. To investigate epigenetically driven ecological stoichiometry we interrogated genomic methylation and fitness in *D. magna* using two separate, but complementary, approaches. This involved supplementation of growth media vitamin B_12_ and MTX, molecules with known effects on OCM.

*D. magna* fitness and global methylation levels differed according to vitamin B_12_ availability and exposure to MTX. Vitamin B_12_ had a positive effect on fitness over a two week period and was associated with increased genomic methylation after only 24 h; while MTX was detrimental to fitness and exposure resulted in loss of DNA methylation. Vitamin B_12_ had a similar positive influence on fitness in *D. magna* to that observed previously in *Daphnia pulex* ^28^, however the underlying molecular mechanisms in this early investigation were unexplored and the effect of the vitamin has not been addressed subsequently. Here, we provide evidence that supports a role for epigenetic mechanisms in *D. magna’s* ability to adapt to their surroundings/dietary composition and convey environmental cues to measurable changes in phenotype. It is indeed plausible that the observed variations of fitness in response to dietary manipulation may be mediated through DNA methylation-dependent changes in gene expression. It is possible that the observed effects are purely correlational and acting via alternative pathways than OCM; however this is unlikely due to the bi-directionality of the effect of manipulation. Therefore, further high resolution profiling of DNA methylation and gene expression in vitamin B_12_ supplemented and MTX-exposed animals is required to reveal which genes and pathways that are directly involved in the observed effects.

Vitamin B_12_, an important factor in the one-carbon metabolic network, is interlinked with methionine synthase and is involved in the donation of one-carbon units from the folate cycle into the methionine cycle through methylation of homocysteine to methionine^32^. Therefore, given that methionine is the precursor to the universal methyl donor *S*-adenosylmethionine (SAM), required for methylation of DNA, it is plausible to interpret that disruptions to this cycle of events can lead to alterations to genomic methylation levels, as exemplified by the increased levels of DNA methylation observed between vitamin B_12_ supplemented and control animals in the current study. Indeed, this relationship between vitamin B_12_ availability and DNA methylation has been reported previously in vertebrates^48^, but not with dramatic changes to fitness. Additionally, we observed that global DNA methylation was reduced following exposure of *D. magna* to a low-dose concentration of MTX. Interpretation of this finding is that the antifolate, MTX (a dihydrofolate reductase inhibitor^49^), inhibits SAM production through reduction of methionine adenyltransferase (MAT) activity^33^, thus limiting the availability of methyl groups that are required for establishing and maintaining genomic methylation. This is consistent with previous findings showing that low-dose MTX induced DNA demethylation in human osteosarcoma cells^50^. Furthermore, a recent study by Blatch *et al*. in *Drosophila* showed that several metabolites, associated with OCM (cystathionine, methylgycine, and methylmalonic acid), were disrupted in larvae consuming MTX^51^; providing evidence that MTX may affect OCM in invertebrates.

Our results demonstrate that potential perturbations of one-carbon metabolism, elicited through vitamin B_12_ deficiency or MTX exposure, affect not only the fitness of the animals but also impact their epigenome. These findings may have a wider context in the natural environment and the growth rate hypothesis (GRH). The GRH states that higher growth rate (μ) is related to higher phosphorus concentration and lower carbon∶phosphorus and nitrogen∶phosphorous ratios^52^ but it implies that any limitation might have an effect on an organisms growth. For instance, a diet deficient in vitamin B_12_ resulted in non-viable eggs in *Blattella germanica*, suggesting that it may affect the growth of some insects (Gordon, 1959). If vitamin availability can influence global DNA methylation and this can drive organism growth, as observed in the current study, then this phenomenon sheds new light on undiscovered mechanisms of growth regulation in these invertebrates; and calls for urgent verification of these findings within the natural habitat. Our work may also be applied to investigating the possible molecular mechanisms involved in the contentious area of transgenerational epigenetic inheritance research^53^. Broadly defined, transgenerational epigenetic inheritance is the transmission of heritable non-DNA-based information (i.e. DNA methylation, histone marks and non-coding RNA molecules) from one generation to the next, through multiple generations^54^. Indeed, intergenerational inheritance has been previously demonstrated in a zinc exposure study in *D. magna*, associated with DNA hypomethylation in non-exposed generations that were linked with changes in gene transcription^47^. In another study, *Daphnia* mothers fed a low phosphorous diet produced smaller neonates with lower body P content compared to control (P-rich) mothers^55^. However, the underlying molecular mechanisms were not interrogated and epigenetic mechanisms represent an attractive possibility to potentially explain some of the observed variation. The use of *Daphnia* to investigate transgenerational epigenetic inheritance is enticing – given their clonal nature and genetic identicalness, thus permitting the separation of genetic and epigenetic influences on phenotype^5^.

It has recently been reasoned that invertebrates (specifically insects) are suitable organisms for studying human diseases with an epigenetic component, due to conservation of underlying molecular mechanisms^56^. Additionally, despite being vital for development in non-mammalian species the mechanisms of vitamin function are poorly understood in invertebrates^51^. The observations outlined in our investigation, showing a link between vitamin B_12_ availability or MTX exposure and levels of DNA methylation, provide novel information on nutritional mechanisms in a non-mammalian species. Our results provide supporting evidence for the use of invertebrate species, particularly *D. magna*, as models for studying the molecular mechanisms involved in environmental epigenetics and transgenerational epigenetic inheritance. These findings also suggest that one carbon metabolism may be limited by vitamins in the natural environment; and we propose that future experiments in natural environments are performed to test this hypothesis. Further experiments are also required to uncover whether B_12_ supplementation or MTX exposure directly affect OCM in invertebrates. Finally, the results of our study show promise for determining whether chronic outcomes of environmental insults are associated with perturbed DNA methylation; using a reliable, cost-effective and rapid assay developed for this purpose.

### Availability of data and material

Should the manuscript be accepted, the data supporting the results will be archived in an appropriate public repository such as Dryad or Figshare and the data DOI will be included at the end of the article.

## Electronic supplementary material

Supplementary figures and tables
