# Supplementary material for: Bi-directional effects of vitamin B12 and methotrexate on Daphnia magna fitness and genomic methylation
Source: Sci Rep. 2017 Sep 19;7:11872. doi: 10.1038/s41598-017-12148-2 (PMC5605502; doi:10.1038/s41598-017-12148-2)
Supplement: Supplementary file 1 — Supplementary figures and tables [file 41598_2017_12148_MOESM1_ESM.pdf]

Supplementary material for

Bi-directional effects of vitamin B_{12} and methotrexate on *Daphnia magna* fitness and genomic methylation

Running title: **Invertebrate model for environmental epigenetics**

Fitore Kusari [1] (fitorekusari@gmail.com), Alan M. O'Doherty [2] (alan.odoherty@ucd.ie), Nikolas J. Hodges [1] (n.hodges@bham.ac.uk) and Marcin W. Wojewodzic #[1] (M.Wojewodzic@bham.ac.uk)

# Corresponding author: Marcin W. Wojewodzic, University of Birmingham, M.Wojewodzic@bham.ac.uk, Tel: +44 (0) 121 415 8200, Fax: +44 (0) 121 414 6056

[1]*University of Birmingham, School of Biosciences, Edgbaston, Birmingham, UK, B15 2TT.*

[2]*School of Agriculture & Food Science, University College Dublin, Belfield, Dublin 4, Ireland.*

Supplementary Table 1 Summary statistics of generalized linear model testing the effects of vitamin B_{12} treatments on fitness of *Daphnia magna*

	Value	Standard Error	Degrees of Freedom	t-value	p-value
Intercept	0.310	0.00048	75	0.004	≤ 0.001
Treatment (B_{12})	0.039	0.0027	75	14.45	≤ 0.001

Generalized linear model (glm) was used to study the relationship between fitness and vitamin B_{12} exposure. For every 1 mg/L media increase of vitamin B_{12}, the fitness of *D.magna* increased by 0.039. The results revealed by glm are statistically significant ($P \leq 0.001$).

Supplementary Table 2 Summary statistics of the impact of vitamin B_{12} on brood size

	Estimate	Std Error	t value	Pr(>|t|)
Intercept	33.12	1.65	20.11	<<0.001
Treatment (**B_{12}**)	17.91	0.95	18.93	<<0.001

For every mg/L of vitamin B_{12} added to the media, the number of offspring increased by 17 compared to a control lacking vitamin B_{12} in media.

Supplementary Table 3 Summary statistics of generalized linear model testing the effects of MTX exposure on *Daphnia magna* fitness

	Value	Standard Error	Degrees of Freedom	t-value	p-value
Intercept	0.38	0.00059	75	0.0059	≤ 0.001
Treatment	-0.002	0.0002	75	-9.58	≤ 0.001

(Methotrexate)

MTX treatment significantly reduced fitness in *D.magna* by 0.002 for every 1 nM increase of MTX in the media ($P \leq 0.001$).

Supplementary Table 4 Summary statistics of the impact of MTX on brood size

	Estimate	Std Error	t value	Pr(>|t|)
Intercept	72.0852	3.08	23.43	<< 0.001
Treatment (**MTX**)	-0.8985	0.11	-8.02	<<0.001

MTX treatment significantly reduced brood size (first and second brood combined) of all exposed *D.magna* mothers. For every 10 nM increase of MTX in the media, the number of offspring decreased by 8.9 compared to the control.

Supplementary figure 1
Impact of B12 exposure on Daphnia magna on first, second and total brood size.

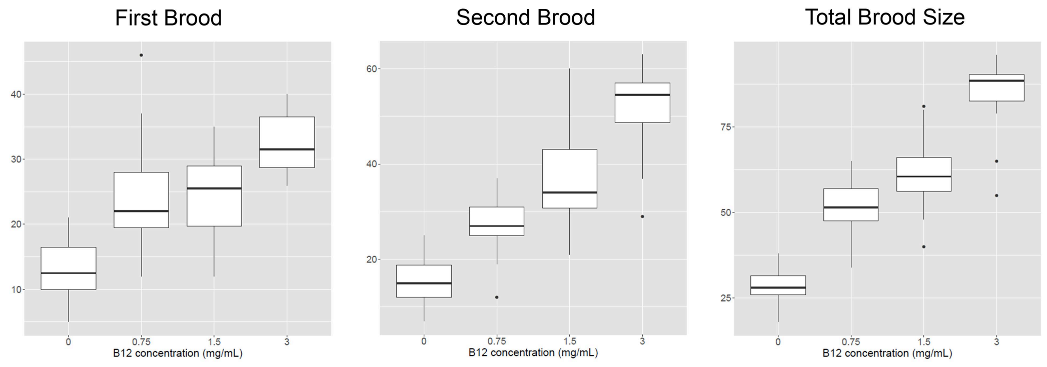

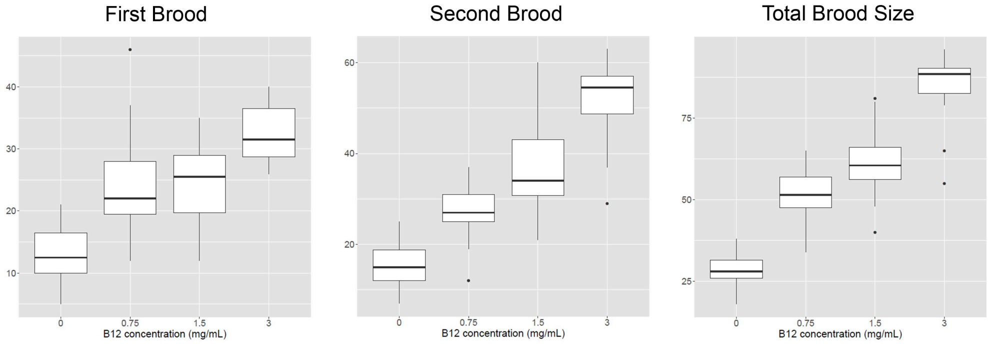

Supplementary Figure 2 Impact of MTX exposure on Daphnia magna on first, second and total brood size.

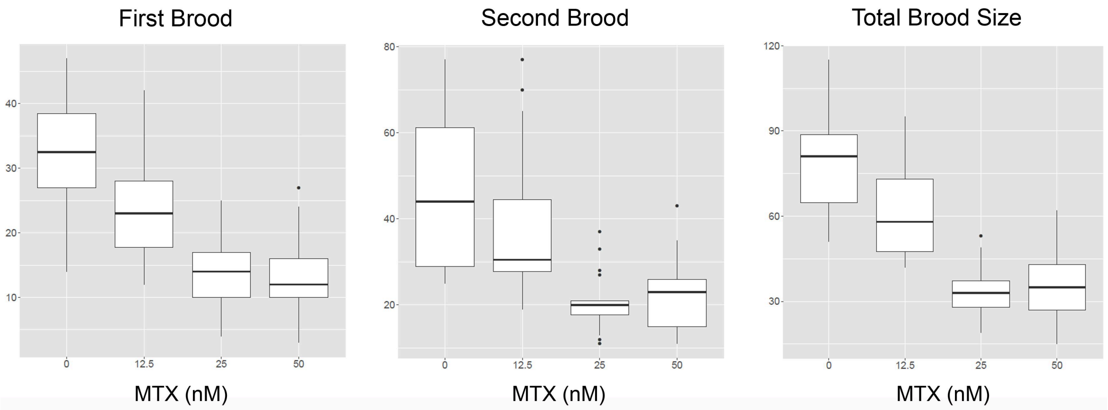

Supplementary Figure 3 The global methylation status of mammalian skin cells (48.7 ± 1.2 %) surpass that of non-mammlian water fleas (Table 5, main text). Amongst Daphnia, animals supplemented with vitamin B12 display the highest level of CpG methylation (9.09 ± 2.4 %) compared to MTX-treated animals and untreated controls (Table 5, main text). A decreasing trend in global methylation levels is observed in Daphnia lacking vitamin B12 and exposed to MTX.

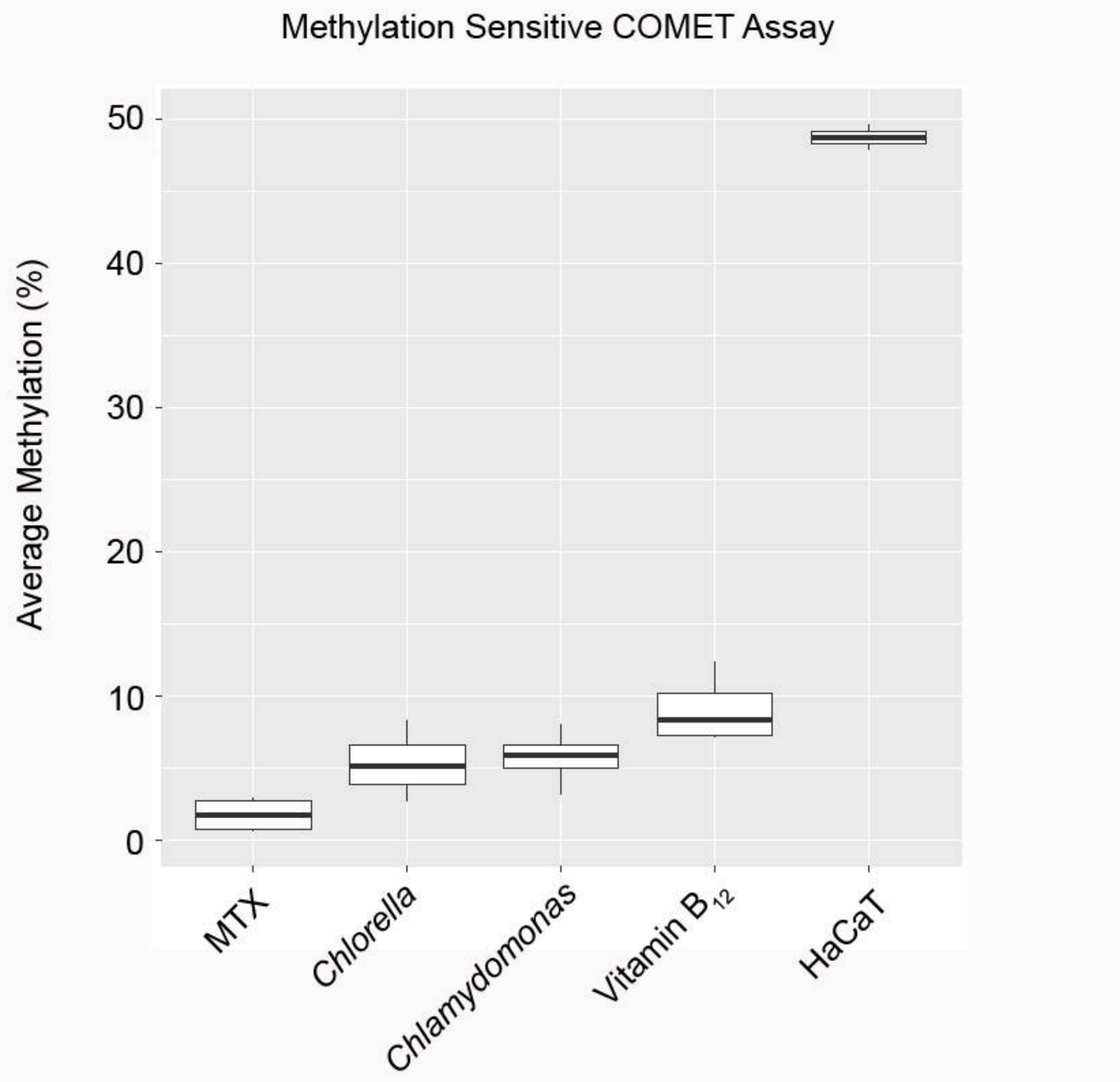

Methylation Sensitive COMET Assay
Average Methylation (%)
50
40
30
20
10
0
MTX
Chlorella
Chlamydomonas
Vitamin B12
HaCaT
